# Two-Dimensional Porous Beryllium Trinitride Monolayer as Multifunctional Energetic Material

**DOI:** 10.3390/nano15131004

**Published:** 2025-06-29

**Authors:** Jiaxin Jiang, Qifan Hu, Weiyi Wang, Hongyan Guo

**Affiliations:** 1 Department of Physics, Anhui Normal University, Wuhu 241000, China; jiangjiaxin@ahnu.edu.cn; 2 Xuancheng Ecological Environment Law Enforcement Monitoring Station, Xuancheng 242000, China; hqf0123456789@sina.com; 3 Hefei National Laboratory for Physical Sciences at Microscale, Department of Chemical Physics, University of Science and Technology of China, Hefei 230026, China

**Keywords:** DFT, polynitrogen compounds, energetic materials, 2D materials

## Abstract

Polynitrogen compounds have broad applications in the field of high-energy materials, making the exploration of two-dimensional polynitride materials with both novel properties and practical utility a highly attractive research challenge. Through global structure search methods and first-principles theoretical calculations at the Perdew–Burke–Ernzerhof (PBE) level of density functional theory (DFT), the globally minimum-energy configuration of a novel planar BeN_3_ monolayer (tetr-2D-BeN_3_) is predicted. This material exhibits a planar quasi-isotropic structure containing pentagonal, hexagonal, and dodecagonal rings, as well as “S”-shaped N6 polymeric units, exhibiting a high energy density of 3.34 kJ·g^−1^, excellent lattice dynamic stability and thermal stability, an indirect bandgap of 2.66 eV (HSE06), high carrier mobility, and ultraviolet light absorption capacity. In terms of mechanical properties, it shows a low in-plane Young’s stiffness of 52.3–52.9 N·m^−1^ and a high in-plane Poisson’s ratio of 0.55–0.56, indicating superior flexibility. Furthermore, its porous structure endows it with remarkable selectivity for hydrogen (H_2_) and argon (Ar) gas separation, achieving a maximum selectivity of up to 10^23^ (He/Ar). Therefore, the tetr-2D-BeN_3_ monolayer represents a multifunctional two-dimensional polynitrogen-based energetic material with potential applications in energetic materials, flexible semiconductor devices, ductile materials, ultraviolet photodetectors, and other fields, thereby expanding the design possibilities for polynitride materials.

## 1. Introduction

High-energy-density materials are crucial in fields such as propellants, explosives, and rocket fuels due to their ability to store exceptionally high energy [1]. Polynitrogen compounds are considered ideal candidates for such materials, primarily because the N≡N triple bond energy in nitrogen gas (N_2_) (954 kJ·mol^−1^) is significantly higher than that of N-N single bonds (~160 kJ·mol^−1^) and N=N double bonds (~418 kJ·mol^−1^) [2]. This allows for the release of enormous energy when N-N/N = N bonds break to form N≡N bonds. Various polynitrogen structures have been investigated, including the cubic gauche phase [3], layered polymeric phases [4], black phosphorus-like structures [5], adamantane-type N_10_ [6], P4/nbm phase [7], and ring/chain/cage configurations [8,9,10]. Alkaline (earth) metal polynitrogen compounds (e.g., MN_3_, MN_5_, MN_10_, MN_4_; M = Li, Na, K, Rb, Cs, Be, Mg, Ca, Sr, Ba, etc.) [11,12,13,14,15,16,17,18,19,20,21,22,23,24,25,26] often contain abundant polymeric nitrogen networks, such as N_3_/N_5_/N_6_ rings or N_∞_ chains [11,14,16,26]. Among them, beryllium (Be)-based polynitrogen compounds exhibit unique advantages in energy density due to their smallest atomic mass and radius when forming compounds with polymeric nitrogen. For instance, the most stable γ-BeN_4_ features a chair-like nitrogen chain structure and exhibits an energy density of 3.1 kJ·g^−1^ [27]. High-pressure-synthesized β-BeN_4_ (obtained from the reaction of Be_3_N_2_ and N_2_ at 25.4 GPa) demonstrates an even higher energy density of 3.6 kJ·g^−1^ while retaining the same chair-like nitrogen chain configuration [28]. Notably, δ-BeN_4_ [27] and P2_1_/c-BeN_4_ [29] exhibit exceptionally high energy densities of 4.7 kJ·g^−1^ and 6.3 kJ·g^−1^, respectively. These values significantly surpass those of alkaline earth metal nitrides such as P1-MgN_4_ (2.084 kJ·g^−1^) [30] and P1-CaN_10_ (2.438 kJ·g^−1^) [31] and even exceed that of the conventional explosive TNT (4.3 kJ·g^−1^). Furthermore, theoretical predictions suggest that α-BeN_6_ and β-BeN_6_ [32] possess high energy densities of 3.32 kJ·g^−1^ and 3.59 kJ·g^−1^, respectively. Therefore, the development of novel high-energy-density materials based on the beryllium–nitrogen (Be-N) system is a highly promising and important direction.

Two-dimensional (2D) materials have been extensively studied, exhibiting various and novel physical and chemical properties with wide applications [33,34,35,36]. In contrast, 2D polynitrogen materials is still in its early stages, that only some 2D polynitrogen materials have been reported, such as, 2D KN_3_ [37], *h*-MN_2_ (M = Be, Mg) [38], *h*-MN_3_ (M = Be, Ge) [39], and 2D MN_4_ (M = Be, Mg, Ir, Rh, Ni, Cu, Au, Pd, Pt) [40]. Similar to bulk polynitrogen compounds, these 2D materials also contain diverse polymeric nitrogen structures, such as N_3_ trimers (2D KN_3_) [37], N_4_ tetramers (*h*-MgN_2_) [38], N_6_ hexagons (*h*-BeN_3_) [39], and infinite N_∞_ chains (2D MgN_4_) [20]. For the 2D Be-N system, many 2D N-rich beryllium polynitrogen compounds have been intensively explored, e.g., BeN_4_, BeN_3_, BeN_2_, Be_2_N_3_, Be_3_N_4_, BeN, and Be_3_N_2_, revealing rich properties and broad application prospects [22,38,41,42]. For example, *h*-BeN_2_ monolayer [38], featuring isolated “Y”-shaped N_4_ tetramers, is predicted to possess a direct bandgap, excellent carrier mobility, ultrahigh on-current in transistors, and water photocatalysis capability. Furthermore, α-2D-BeN_2_ consists of penta-, hexa-, and hepta-atomic rings, along with an N_4_ tetramer, currently the most stable 2D BeN_2_ structure, theoretically exhibits a direct bandgap of 1.82 eV, and demonstrates outstanding performance in oxygen reduction/evolution reaction catalysis and potassium-ion storage [43]. A benzene-like N_6_ hexagonal honeycomb monolayer material, *h*-BeN_3_, shows high stability, excellent carrier mobility, and *n*-type semiconductor properties, making it promising for nanoelectronic device applications [39]. In addition, recent studies have achieved multiscale correlation from atomic mechanisms to mesoscopic morphology in group III nitride nanosystems by coupling DFT with phase-field modeling, establishing a theoretical foundation for predicting broader semiconductor nanostructures and advancing the development of novel nitrogen-containing compounds [44]. Experimentally, the successful synthesis of a 2D BeN_4_ monolayer with parallel armchair-like infinite [N]_n_ chains possesses anisotropic characteristics and potential applications in ion battery storage, CO_2_ capture, and hydrogen storage [22]. Nevertheless, searching for more practical 2D polynitrogen materials with novel, advanced properties is in the ascendant.

In this work, using the artificial bee colony (ABC) algorithm for 2D global structure search, a novel tetragonal porous BeN_3_ monolayer (tetr-2D-BeN_3_), consisting of Be atoms and “S”-shaped N_6_ tetramer segments, is systematically investigated. Compared with *h*-BeN_3_ [39], it has lower energy while maintaining excellent lattice dynamics and thermal stability, along with a high energy density of 3.34 kJ·g^−1^. This new structure exhibits an indirect bandgap (2.66 eV) with high carrier mobilities, strong ultraviolet light absorption, and a large-pore structure that enables superior gas separation performance. It is expected to serve as a highly stable multifunctional material with broad application prospects in various fields.

## 2. Materials and Methods

### 2.1. Materials

The artificial bee colony (ABC) algorithm within the CALYPSO code [45] is employed to conduct a global structural search for two-dimensional (2D) compounds with a Be:N = 1:3 (BeN_3_) stoichiometric ratio. This study focuses on the lowest-energy structure from these 1800 candidates, a 2D planar BeN_3_ structure with *P4m* 2D space group (wallpaper group).

### 2.2. Calculation Details

First-principles simulations based on density functional theory (DFT) [46,47] are performed using the VASP (vesion 6) software package [48]. The electron–ion interactions are described by the projector augmented wave (PAW) method [49], with a plane-wave basis set energy cutoff of 520 eV. The exchange–correlation effects are treated using the Perdew–Burke–Ernzerhof (PBE) correlation–exchange (XC) functional. For geometric optimization and electronic property calculations, the Γ-centered k-point mesh in the Brillouin zone is set to 6 × 6 × 1. The energy convergence threshold is set to 10^−5^ eV, and the atomic force convergence threshold is 0.01 eV/Å. All intrinsic pristine structures are optimized using the ISIF = 3 tag in VASP, where stress convergence is automatically controlled by the force convergence criterion. The more accurate electronic structure is obtained using the Heyd–Scuseria–Ernzerhof (HSE06) hybrid functional [50]. The lattice dynamical stability of the material is verified through phonon spectra calculations using the finite displacement method [51]. Ab initio molecular dynamics (AIMD) simulations are conducted in the NVT ensemble with a time step of 1 fs for a total duration of 5 ps to evaluate the structural stability at elevated temperatures. The structural search within the CALYPSO code [45] settings included parallel calculations for unit cells with varying numbers of atoms, specifically examining 2:6, 3:9, and 4:12 configurations. All calculations use a population size of 20 and 30 generations of evolution, generating a total of 1800 candidate structures.

The formation energy of tetr-2D-BeN_3_ is calculated using the following equation:

$$E_{f}=\left(E_{BeN_{3}}-E_{Be}-3E_{N}\right)/4$$

Here, $E_{Be}$ and $E_{N}$ represents the energy of per metal atoms in the Be bulk metal phase and the energy of per atoms in the N_2_ gas.

The vacancy formation energies (*E*_F-vac_) are calculated using the following equation:

$$E_{\text{F-vac}}=E_{V_{Be}/V_{N}@BeN_{3}}+E_{B\text{e-}atom/\text{N-}atom}-E_{BeN_{3}}$$

Here, $E_{B\text{e-}atom}$ and $E_{\text{N-}atom}$ represents the energy of a single Be and N atom.

The in-plane Young’s stiffness Y(θ) and Poisson’s ratio v(θ) are calculated as functions of θ based on the equations as listed below [52]:

$$Y\left(\theta \right)=\frac{C_{11}C_{22}-{C_{12}}^{2}}{C_{11}\sin^{4}\theta +C_{22}\cos^{4}\theta +(\frac{C_{11}C_{22}-{C_{12}}^{2}}{C_{66}}-2C_{12})\cos^{2}\theta \sin^{2}\theta }$$

$$v\left(\theta \right)=\frac{(C_{11}+C_{12}-\frac{C_{11}C_{22}-{C_{12}}^{2}}{C_{66}})\cos^{2}\theta \sin^{2}\theta -C_{12}\sin^{4}\theta -C_{12}\cos^{4}\theta }{C_{11}\sin^{4}\theta +C_{22}\cos^{4}\theta +(\frac{C_{11}C_{22}-{C_{12}}^{2}}{C_{66}}-2C_{12})\cos^{2}\theta \sin^{2}\theta }$$

According to the DP theory [53], the carrier mobility of a 2D structure is calculated as

$$\mu_{2D}=\frac{e\hbar^{3}C_{2D}}{k_{B}Tm^{*}m_{d}{\left(E_{1}\right)}^{2}}$$

where e, ℏ, and $k_{B}$ are the electron charge, reduced Planck constant, and Boltzmann constant, respectively. $C_{2D}$ are the elastic moduli, T is the temperature (300 K), $m^{*}$ is the effective mass along the transport direction, which can be obtained by fitting the band structure at the CBM and VBM, and $m_{d}=\sqrt{m_{x}^{*}m_{y}^{*}}$ is the average effective mass. $E_{l}$ is the deformation potential constant, which is the key to the magnitude of mobility, defined by $E_{l}=\partial E_{edge}/\partial \varepsilon$, where $E_{edge}$ is the energy of the band edge and $\varepsilon =∆l/l_{0}$. The strain range is from −0.6% to +0.6% with an interval of 0.2%.

According to the frequency-dependent permittivity of $\varepsilon \left(\omega \right)=\varepsilon_{1}\left(\omega \right)+i\varepsilon_{2}\left(\omega \right)$, the absorption coefficient is obtained based on the following equation [54,55]:

$$\alpha \left(\omega \right)=\frac{\sqrt{2}\omega }{c}{\left[\sqrt{\varepsilon_{1}^{2}\left(\omega \right)+\varepsilon_{2}^{2}(\omega )}-\varepsilon_{1}(\omega )\right]}^{1/2}$$

The selectivity $S_{gas1/gas2}$ is an important indicator of the efficiency of gas separation and can be assessed with the Arrhenius equation [56]:

$$S_{gas1/gas2}=\frac{r_{gas1}}{r_{gas2}}=\frac{A_{gas1}}{A_{gas2}}\frac{e^{-E_{gas1}/RT}}{e^{{-E}_{gas2}/RT}}$$

where *r* is the diffusion rate, *A* is the diffusion prefactor (assuming that the *A* of gas molecules are the same as 10^11^) [57], *E* is the diffusion energy barrier, *R* is the gas constant, and *T* is the temperature.

## 3. Results and Discussion

### 3.1. Structural Search and Energetic Properties of BeN_3_

**Figure 1:**
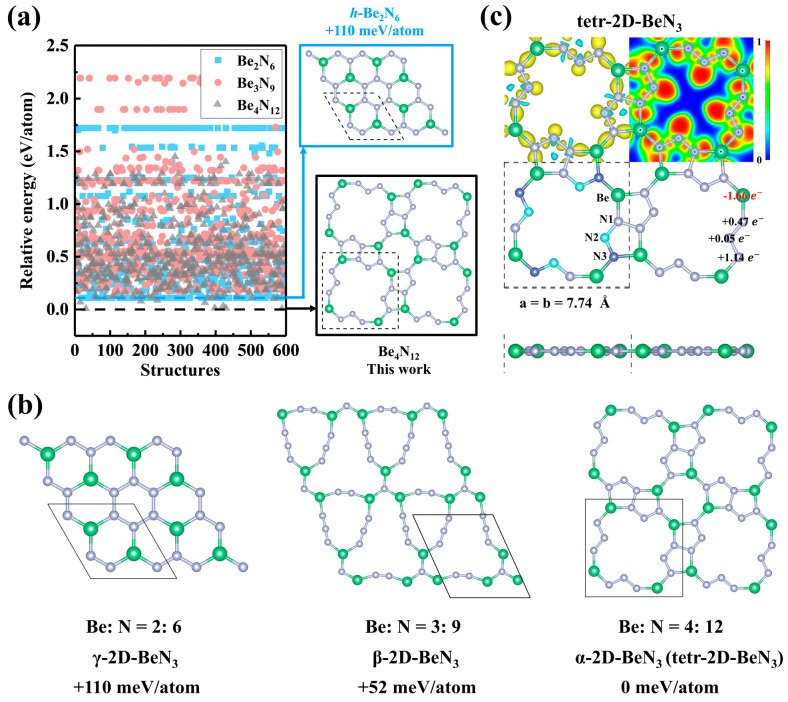
(**a**) Global structural search results for Be:N = 1:3, where the blue box indicates the previously reported 2D Be-N monolayer, and the black box marks the global energy-minimum 2D Be-N monolayer. (**b**) The top and side views of the calypso structure search for the lowest-energy configurations of Be:N ratios 2:6, 3:9, and 4:12. The structures are labeled as α/β/γ-2D-BeN_3_ in ascending order of energy. (**c**) The top and side views, deformation charge density, electron localization function, and Bader charge analysis of tetr-2D-BeN_3_.

First-principles calculations reveal that the global energy-minimum configuration of BeN_3_ is found in the largest unit cell system (containing 4 Be atoms and 12 N atoms) (Figure 1a,b), while previously reported monolayer structure (*h*-Be_2_N_6_) [39] with the same stoichiometry are identified in the corresponding smallest unit cells. This result suggests that increasing the unit cell size facilitates the discovery of global energy-minimum structures for two-dimensional (2D) Be-N compounds, providing a new strategy for global structural searches of similar 2D materials.

As shown in Figure 1a, the global energy-minimum 2D-Be_4_N_12_ (here named tetr-2D-BeN_3_) exhibit significant thermodynamic advantages over the reported *h*-Be_2_N_6_ [39], with a total energy reduction of 110 meV/atom. The tetr-2D-BeN_3_ monolayer possesses a tetragonal orthogonal lattice with lattice constants *a* = *b* = 7.74 Å and a space group (wallpaper group in 2D) symmetry of *P4m*. As shown in Figure 1b, the unit cell contains four equivalent Be atoms and three types of nitrogen atoms (N1, N2, N3), where N1 adopts a Be-N-N tri-coordination mode, N2 exhibits N-N di-coordination, and N3 shows Be-Be-N tri-coordination. The tetr-2D-BeN_3_ planar structure consists of five-membered, eight-membered, and twelve-membered rings, featuring an “S”-shaped N_6_ cluster. The Be-N bond lengths range from 1.61 to 1.65 Å, while the N-N bond lengths (1.32–1.41 Å) lie between the typical single N–N bond (1.45 Å) [58] and double N=N bond (1.25 Å) values [59], suggesting its potential as an energetic material. Under ambient pressure, the exothermic decomposition of BeN_3_ yields the most stable Be-N compound Be_3_N_2_ ($Ia\bar{3}$) [28] and N_2_, as shown in the following equation:

$$6BeN_{3}\to {2Be}_{3}N_{2}+7N_{2}+10.6\;eV$$

This process releases a chemical energy of 10.6 eV, corresponding to an energy density of approximately 3.34 kJ·g^−1^, which is slightly lower than that of BeN_4_ (3.60 kJ·g^−1^) [28], δ-BeN_4_ (4.70 kJ·g^−1^) [27], β-BeN_6_ (3.59 kJ·g^−1^) [32], and HEDM TNT (4.30 kJ·g^−1^) but higher than CNO (2.2 kJ·g^−1^) [60], LiN_5_ (2.72 kJ·g^−1^) [61], GdN_6_ (1.62 kJ·g^−1^) [62], YN_10_ (3.05 kJ·g^−1^) [63], γ-BeN_4_ (3.10 kJ·g^−1^) [27].

### 3.2. Bonding Characteristics and Stability of BeN_3_

To further elucidate the bonding characteristics in tetr-2D-BeN_3_, deformation charge density, electron localization function (ELF), and Bader charge analysis are calculated (Figure 1c). The deformation charge density reveals electron accumulation at Be-N bridging sites and partial electron distribution between adjacent nitrogen atoms within the N_6_ chain segment, indicating significant covalent hybridization between Be-N atoms and within the N_6_ chains. Additionally, significant electron density is observed on the N2 surface along the direction opposite to the N1-N2-N3 angle bisector. Analysis of the ELF (where high values (>0.5) correspond to lone pairs, core electrons, or covalent bonds, low values (<0.5) represent ionic bonds, and ELF~0.5 signifies metallic bonding) shows a highly localized electron cloud (ELF ≈ 1) on the N2 surface, indicating the presence of unhybridized *p*-orbitals along the direction normal to the plane. The high ELF values at Be-N and N-N sites further suggest the formation of strong covalent bonds. Bader charge analysis quantifies electron transfer, revealing that Be atoms transferred 1.66 e^−^, while nitrogen atoms exhibited significant charge variation. Specifically, the tri-coordinated N1 (Be-N-N), di-coordinated N2 (N-N), and tri-coordinated N3 (Be-Be-N) acquired charges of +0.47 e^−^, +0.05 e^−^, and +1.14 e^−^, respectively. Crucially, the notably low charge acquisition and the high ELF localization on the di-coordinated N2 atom, indicative of poorly hybridized/underbonded states due to its low coordination and minimal charge transfer, are highly pertinent to the observed high energy density of tetr-2D-BeN_3_. The electron-rich N2 center, featuring unpaired electrons or highly localized electrons within its *p*_z_ orbital with a lone pair electron perpendicular to the plane, represents a significant metastable site storing considerable strain energy. Upon decomposition or reaction initiation, these energetically frustrated sites, particularly the under-bonded and electron-deficient N2, provide a substantial thermodynamic driving force, facilitating the rapid release of stored chemical energy and thereby underpinning the material’s high energy density.

**Figure 2:**
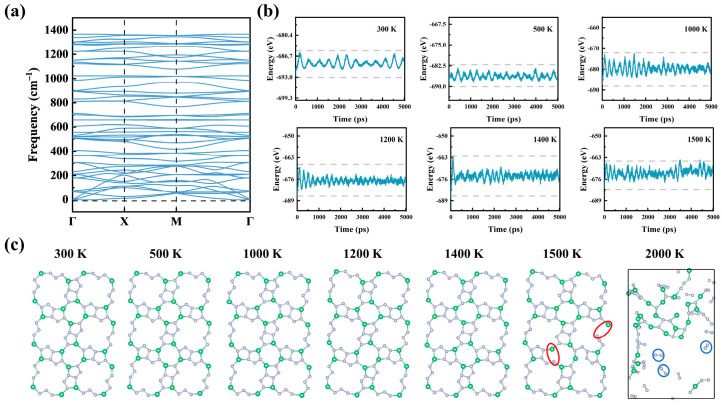
(**a**) Phonon spectrum of tetr-2D-BeN_3_, (**b**) the energy curves, and (**c**) final structural snapshots of tetr-2D-BeN_3_ from AIMD simulations at 300, 500, 1000, 1200, 1400, and 1500 K over 5 ps, as well as structural snapshots from AIMD simulations at 2000 K. The red circles indicate broken Be-N bonds, while the blue circles highlight N_2_ molecules formed upon the decomposition of tetr-2D-BeN_3_.

**Figure 3:**
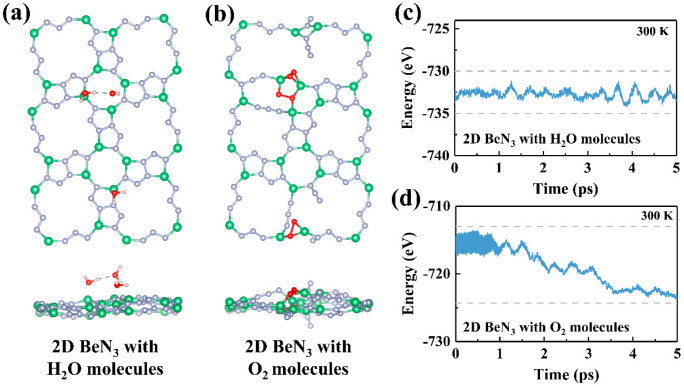
The structural snapshots (**a**,**b**) and the energy curves (**c**,**d**) of tetr-2D-BeN_3_ with H_2_O and O_2_ molecules from AIMD simulations at 300 K over 5 ps.

Furthermore, the stability of the novel tetr-2D-BeN_3_ monolayer is evaluated from three perspectives: structural stability, thermodynamic feasibility, and dynamical stability. The formation energy (*E_f_*, referenced to Be metal and N_2_ molecules) is calculated as +0.02 eV/atom, suggesting that the formation of tetr-2D-BeN_3_ from Be metal and N_2_ molecules requires slight external energy input. Phonon spectrum analysis (Figure 2a) confirms the dynamical stability of tetr-2D-BeN_3_, as no imaginary frequencies are observed across the entire Brillouin zone. Finally, AIMD simulations verify the thermal stability of this new structure. Figure 2b displays geometric snapshots after 5 ps of simulation at temperatures of 300, 500, 1000, 1200, and 1400 K, showing that the tetr-2D-BeN_3_ monolayer maintains structural integrity below 1400 K, exhibits partial bond breaking at 1500 K. At 2000 K, tetr-2D-BeN_3_ completely decomposes into amorphous Be-N clusters and N_2_ molecules. This theoretical prediction of decomposition temperatures (2000 K) is higher than the experimentally measured decomposition temperatures of graphene and MoS_2_ monolayers (1100–1300 K) [64,65], and serves as a reference for comparative thermal stability analysis. Furthermore, the environmental stability of tetr-2D-BeN_3_ through AIMD simulations at 300 K under both H_2_O and O_2_ atmospheres is investigated. As shown in Figure 3, the tetr-2D-BeN_3_ monolayer demonstrated excellent structural stability during 5 ps AIMD simulations in H_2_O environment, with energy fluctuations remaining within a stable range. However, in an O_2_ environment, significant oxidation is observed, characterized by the formation of localized Be-O compounds and the cleavage of Be-N bonds. These results, combined with vacuum stability tests, demonstrate that while tetr-2D-BeN_3_ maintains thermal stability up to 1400 K in vacuum, it shows pronounced oxygen sensitivity at ambient conditions, necessitating oxygen-free environments during both preparation and application.

**Figure 4:**
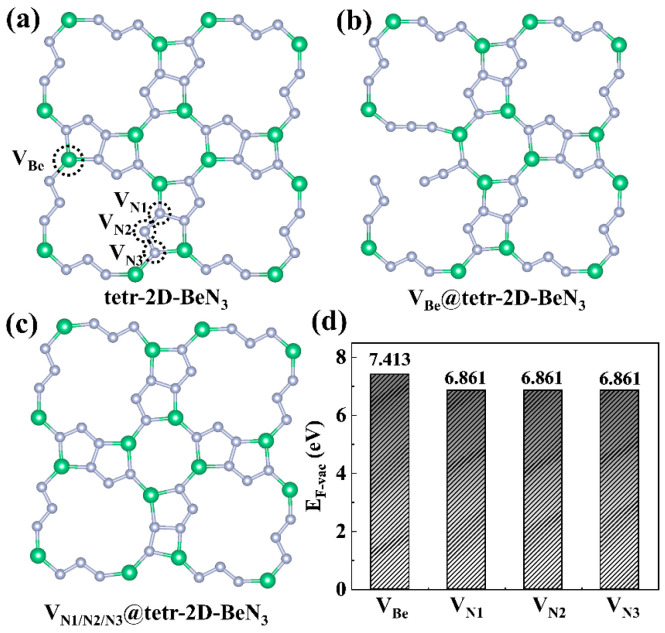
(**a**) Four different types of point vacancies on the tetr-2D-BeN_3_ surface, along with the fully optimized structures of (**b**) V_Be_@tetr-2D-BeN_3_ and (**c**) V_N1/N2/N3_@tetr-2D-BeN_3_, as well as their corresponding (**d**) vacancy formation energies.

Considering the tendency for point vacancies to form during the synthesis of 2D materials, four different types of point vacancies on the tetr-2D-BeN_3_ surface are tested, labeled as V_Be_, V_N1_, V_N2_, and V_N3_ (Figure 4a). As shown in Figure 4b,c, after fully structural optimization, the V_Be_ vacancy is found to cause structural disruption in the tetr-2D-BeN_3_ sheet, while the structures with V_N1_, V_N2_, and V_N3_ vacancies only transform the original Be-N pentagonal rings into Be-N quadrilateral rings without significant changes to the overall crystal structure. Furthermore, calculations of vacancy formation energies (E_F-vac_) reveal that the E_F-vac_ values for V_Be_ and V_N1/N2/N3_ are 7.413 eV and 6.861 eV, respectively, indicating that V_Be_ is more difficult to form than V_N_ (Figure 4d). Additionally, E_F-vac_ exceeding 5 eV suggests that low-density vacancies are energetically unlikely to form on the tetr-2D-BeN_3_ surface. Furthermore, considering that many nitrides require high-pressure conditions for synthesis and that tetr-2D-BeN_3_ additionally possesses porous characteristics, the experimental synthesis of this material still faces significant challenges.

### 3.3. Mechanical, Electronic, and Optical Properties of BeN_3_

For mechanical properties, the elastic constants of tetr-2D-BeN_3_ are calculated as *C*_11_ = *C*_22_ = 76.2, *C*_12_ = 42.7, and *C*_66_ = 17.0 N·m^−1^ satisfying the mechanical stability criteria for 2D structures (*C*_11_ > 0, *C*_22_ > 0, *C*_11_*C*_22_ − *C*_12_*C*_21_ > 0, *C*_66_ > 0) [66], confirming its mechanical stability. Tetr-2D-BeN_3_ exhibits quasi-isotropic mechanical behavior, the in-plane Young’s stiffness (Y(θ)) is in range of 52.3–52.9 N·m^−1^ with a tiny anisotropy of *Y*_max_/*Y*_min_ = 1.01, indicating a relatively soft in-plane characteristic, comparable to silicene (62 N·m^−1^) [67] and phosphorene (21–91 N·m^−1^) [68] but significantly lower than graphene (330 N·m^−1^) [69] and h-BN (279 N·m^−1^) [70]. The Poisson’s ratio of tetr-2D-BeN_3_ is high (0.55–0.56), reflecting high ductility.

**Figure 5:**
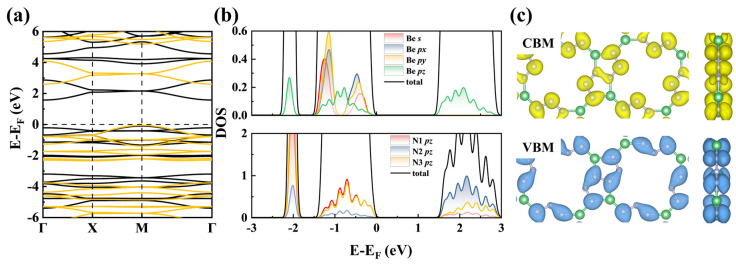
(**a**) The band structures, the black and yellow lines represent the PBE functional and HSE06 functional, respectively, (**b**) projected density of states (PDOS, PBE), and (**c**) partial electron density of band edge of tetr-2D-BeN_3_. The Fermi level is set to 0 eV.

The band structure of tetr-2D-BeN_3_ shown in Figure 5a, reveals an indirect bandgap of 1.68 eV (PBE), with the valence band maximum (VBM) located at the M point (1/2, 1/2, 0) and the conduction band minimum (CBM) at the Γ point (0, 0, 0). HSE06 hybrid functional calculations yielded a more accurate bandgap of 2.66 eV. As shown in Figure 5b,c, projected density of states (PDOS, PBE) and partial charge analysis show that the VBM of tetr-2D-BeN_3_ is dominated by the *p_z_* orbitals of N1 and N3, while the CBM arises from the *p_z_* orbitals of N2 and minor contributions from Be *p_z_* orbitals.

To gain deeper insight into the carrier transport properties of tetr-2D-BeN_3_, deformation potential theory is employed to calculate its carrier mobility. With a tetragonal lattice structure, tetr-2D-BeN_3_ exhibits identical mobility along its two principal axes (a/b directions). For electrons (E) and holes (H), the deformation potentials and effective masses of E/H along these axes are 1.012/0.986 eV and 0.974/0.806 *m_e_*, respectively. The calculated mobilities of E/H reach 1.6/2.5 × 10^3^ cm^2^V^−1^s^−1^, which is lower than those of graphene (340 × 10^3^ cm^2^V^−1^s^−1^) [71] and monolayer black phosphorus (26 × 10^3^ cm^2^V^−1^s^−1^) [72], but higher than that of MoS_2_ monolayer (0.072–0.2 × 10^3^ cm^2^V^−1^s^−1^) [73]. These findings suggest that tetr-2D-BeN_3_ could be a promising candidate for microelectronic applications.

**Figure 6:**
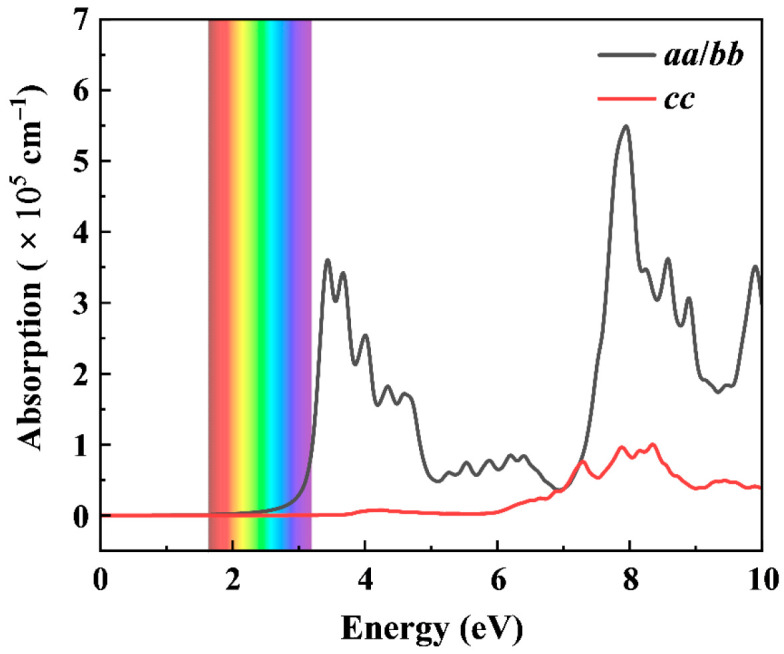
Optical absorption coefficient of tetr-2D-BeN_3_ computed based on the HSE06 method.

The optical properties of tetr-2D-BeN_3_ (calculated at the HSE06 level) reveal anisotropic dielectric constants, satisfying *ε*_aa_ = *ε*_bb_ ≠ *ε*_cc_. As shown in Figure 6, the tetr-2D-BeN_3_ exhibits an indirect bandgap of 2.66 eV and anisotropic optical absorption spectra, with significant differences between the in-plane (*a*/*b*) and out-of-plane (*c*) absorption coefficients. Its absorption in the visible range is weak, with only a gradual increase along the *a*/*b* direction at 2.6 eV. However, in the ultraviolet range, absorption peaks at 3.5 eV and 8 eV, reaching 3.7 and 5.5 × 10^5^ cm^−1^. This unique anisotropic optical response, combined with its 2.66 eV indirect bandgap, suggests that tetr-2D-BeN_3_ holds potential for applications in polarization-sensitive optoelectronic devices and ultraviolet photodetection.

### 3.4. Gas Separation Applications of BeN_3_

**Figure 7:**
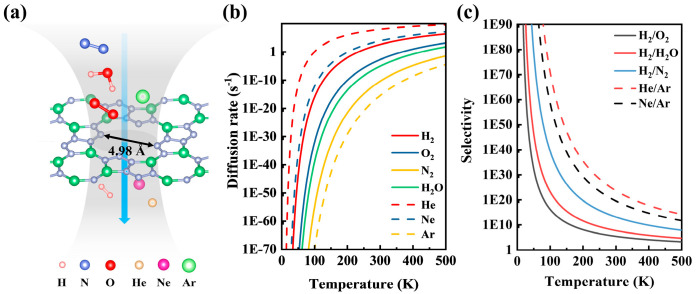
(**a**) Schematic diagram of the tetr-2D-BeN_3_ as a gas separation membrane. (**b**) The diffusion rate and (**c**) selectivity for gas molecules as a function of temperature.

The tetr-2D-BeN_3_ monolayer structure contains twelve-membered rings with a pore diameter of 4.98 Å, indicating its potential for gas separation. Further studies reveal that tetr-2D-BeN_3_ exhibits low energy barriers for the permeation of helium (He), neon (Ne), and hydrogen (H_2_), with values of 0.241 eV, 0.486 eV, and 0.564 eV, respectively. This suggests that He, Ne, and H_2_ can diffuse rapidly across the membrane over a wide temperature range, as illustrated in Figure 7a,b. Moreover, tetr-2D-BeN_3_ demonstrates high selectivity for Ar and H_2_ compared with other noble gases and common atmospheric gases (Figure 7c). Specifically, the selectivity ratios for He/Ar, Ne/Ar, H_2_/O_2_, H_2_/H_2_O, and H_2_/N_2_ reach 10^23^, 10^19^, 10^6^, 10^8^, and 10^13^, respectively. These gas separation capabilities are comparable to those of porous phosphorene and porous silicene [74,75]. Therefore, tetr-2D-BeN_3_ holds significant application value and broad prospects in the field of gas separation.

## 4. Conclusions

In summary, based on first-principles calculations, the energetic properties, structural stability, mechanical properties, electronic properties, optical absorption characteristics, and gas separation performance of the porous tetr-2D-BeN_3_ monolayer are systematically investigated. The tetr-2D-BeN_3_ is a global energy-minimum 2D structure searched by the artificial bee colony (ABC) algorithm. This monolayer material exhibits high energy density of 3.34 kJ·g^−1^, excellent lattice dynamic stability and maintains its fundamental structural framework with outstanding thermal stability up to approximately 1400 K. It demonstrates in-plane quasi-isotropic mechanical, electronic, and optical properties, including a Young’s stiffness of 52.3–52.9 N·m^−1^, a large Poisson’s ratio of 0.55–0.56, a moderate indirect bandgap of 2.66 eV, along with high carrier mobilities of 1.6/2.5 × 10^3^ cm^2^V^−1^s^−1^ and strong ultraviolet light absorption capability. The porous nature of the material enables highly efficient gas separation for hydrogen and argon, achieving maximum selectivity values of 10^13^ (H_2_/N_2_) and 10^23^ (He/Ar), respectively. Owing to these novel characteristics and exceptional performance, the tetr-2D-BeN_3_ monolayer shows potential applications in energetic materials, flexible semiconductor devices, ductile materials, ultraviolet photodetectors, and other fields, thereby expanding the design possibilities for polynitride materials.

## Data Availability

The data that support the findings of this study are available from the corresponding author upon reasonable request.
